# **An approach to using stranding data to monitor cetacean population trends and guide conservation strategies**

**DOI:** 10.1038/s41598-025-12928-1

**Published:** 2025-08-20

**Authors:** Rachel L. Lennon, Rosie S. Williams, Kathryn J. Allan, Mariel T.I. ten Doeschate, Nicholas J. Davison, Simon A. Babayan, Andrew C. Brownlow

**Affiliations:** 1 School of Biodiversity, One Health and Veterinary Medicine, University of Glasgow, Graham Kerr Building, University Avenue, Glasgow, G12 8QQ UK; 2 Centre for Ecology and Conservation, University of Exeter, Penryn Campus, Penryn, Cornwall, TR10 9EZ UK; 3 Institute of Zoology, Zoological Society of London, Regent’s Park, London, NW1 4RY UK

**Keywords:** Cetaceans, Spatiotemporal analysis, Strandings, Opportunistic, Monitoring, Ecology, Conservation biology

## Abstract

Growing anthropogenic pressures increasingly impact marine wildlife, with cetaceans being particularly vulnerable to cumulative effects of stressors due to their position as top predators. As sensors and sentinels of ocean health, cetaceans offer critical insight into known and emerging threats to marine ecosystems. Stranding schemes provide a cost-effective means to assess mortality rates and population demographics, offering insights that are often challenging to obtain through live monitoring. Using a 30-year dataset from the Scottish Marine Animal Stranding Scheme (SMASS) we demonstrate how opportunistically obtained stranding data can be used to monitor populations and guide conservation strategies. Species were clustered into broad ecological groups - baleen whales, short-beaked common dolphins, deep divers, harbour porpoises and pelagic dolphins - for spatiotemporal analysis of stranding patterns. All groups showed increases in annual stranding rates over the study period, with common dolphins and baleen whales exhibiting exponential increases, suggesting these species may be facing heightened pressures. Distinct seasonal and spatial trends were detected, with harbour porpoises predominantly stranding on the east coast and other groups clustering to Scotland’s west coast. Identifying these trends helps focus surveillance and mitigation efforts, underscoring the importance of this approach for monitoring vulnerable species.

**Supplementary Information:**

The online version contains supplementary material available at 10.1038/s41598-025-12928-1.

## Introduction

Growing anthropogenic activity is exerting continuously increasing pressure on the environment, causing significant detrimental effects on wildlife populations^1,2^. In the marine environment, predominant threats include declining fish stocks, increased bycatch and entanglement rates, habitat change from warming sea temperatures, and cascading impacts from chemical, plastic, and noise pollution^3–8^. Robust, long-term monitoring is recognised as a critical first step for understanding the broadscale impacts of these pressures, especially when applied to umbrella species^9^. As top predators, cetaceans are valuable sentinels of wider oceanic health, and changes in their populations often echo changes occurring in lower trophic levels^10^. Overlap in diet and habitat with humans allows marine mammals to serve as early indicators of emerging public health concerns (i.e., bioaccumulation of chemical pollution, new and emerging pathogens) and changes in commercially exploited fish stocks^11^. Marine mammals are also particularly vulnerable to anthropogenic pressures, with many cetacean species experiencing habitat shifts, deteriorations in health, reduced reproductive capacity and ultimately, population declines^12,13^. Monitoring cetaceans is essential for understanding population trends to satisfy international legislation^14,15^, and for assessments of wider ocean health^16,17^.

The intrinsic inaccessibility of the deep ocean makes it exceedingly difficult to monitor, or even detect, population trends in cryptic species^18^. Effort-based surveys of live cetaceans provide population insights, but high financial and logistical costs limit their temporal resolution^19,20^. Stranding schemes are recognised as a cost-effective complement to live-animal monitoring through the collection and collation of biological data and observed mortality rates^21–23^. A significant challenge with stranding data is the opportunistic nature of the datasets, resulting in a biased reporting of mortality^24^. Environmental (e.g., prevailing winds, tide, carcase buoyancy) and reporting factors (e.g., human population density and awareness, popularity of coastline, variations in effort, accurate species ID) influence the likelihood that a stranded animal is found and accurately reported^25^. Thus, it can be difficult to disentangle trends derived from stranding data and determine whether they are a result of biological processes or simply an artefact of the recording and reporting methodology. Despite these challenges, the paucity of viable alternatives for cetacean population monitoring means opportunistic stranding data is a valuable, but often under-utilised, resource.

Globally, numerous schemes collect opportunistic data on stranded marine mammals along local coastlines^26^. The condition of the carcase will dictate the level of data collected, from basic ‘Level A’ data which consists of species, location, and date of stranding, to full post mortem examination^27,28^. Post mortem and morphometric data provides a higher level of information for each case, but also introduces a degree of subjectivity to the data, as interpretation of pathological findings varies by researcher experience^29^. Diagnostic testing and post mortems are also more resource intensive, and collection of this data will vary between schemes^27^. At a minimum, most stranding networks collect ‘Level A’ data and demographic information whenever feasible^27^.While this represents the lowest resolution of data collected from a stranding event, it is both objective and relatively straightforward to gather^28^. As a result, it is the most widely collected data across networks, making it particularly valuable for detecting broad-scale trends in monitored species over time, both within and between stranding networks^27^.

Long-term spatiotemporal data can establish baseline population trends, serving as benchmarks for populations inhabiting or migrating through specific areas^30,31^. Deviations from baselines indicate environmental changes, with the direction and severity of the deviation offering insight into emerging pressures^30^. Further analysis of life history traits, such as age and sex, provide information on population dynamics and help assess species’ resilience to changing environments^32–34^. These patterns can also reveal seasonal periods of vulnerability, such as breeding seasons, underscoring times when conservation efforts should be intensified to mitigate additional anthropogenic stressors^22^. Continued monitoring then allows for assessments of mitigation efforts and can be used to recommend further adjustments where necessary, facilitating an iterative approach to conservation^35^. However, to date, relatively few studies have fully leveraged these low-cost datasets for comprehensive multi-species analyses.

In this study, we present a novel approach to using stranding data as a tool for monitoring cetacean population trends and informing conservation strategies. We analysed a three-decadal dataset from the Scottish Marine Animal Stranding Scheme (SMASS), demonstrating the applicability of opportunistic mortality monitoring networks worldwide. This method enabled the establishment of baseline stranding rates, identification of at-risk species, and highlighted vulnerability in five key cetacean groups: baleen whales, short-beaked common dolphins (*Delphinus delphis*), deep divers, harbour porpoises (*Phocoena phocoena*), and pelagic dolphins. We show how even basic ‘Level A’ strandings data from opportunistic surveillance networks can effectively inform targeted conservation efforts for cryptic yet important species.

## Results

A total of 5147 cetaceans were included in this study as stranding in Scotland between 1992 and 2022. The total number of strandings by group were as follows: baleen whales (*n* = 479, 9%), common dolphins (*n* = 494, 10%), deep divers (*n* = 281, 5%), harbour porpoises (*n* = 2676, 52%), pelagic dolphins (*n* = 1217, 24%) (Table 1). All final models used for analysis can be found in the supplementary materials (Table S1).

**Table 1:** Summary of cetacean stranding groups, associated species and total stranding event counts occurring in Scotland between 1992 and 2022.

Stranding group	Common name of species (number of individuals)	Latin name of species	Count
**Baleen whales**	Minke whale (*n* = 368), humpback whale (*n* = 23), fin whale (*n* = 22), indeterminate baleen whale (*n* = 66)	*Balaenoptera acutorostrata*, *Megaptera novaeangliae*, *Balaenoptera physalus*	479
**Common dolphins**	Short-beaked common dolphin (*n* = 494)	*Delphinus delphis*	494
**Deep divers**	Sperm whale (*n* = 131), Sowerby’s beaked whale (*n* = 56), goose-beaked whale (*n* = 119), northern bottlenose whale (*n* = 34), indeterminate beaked whale (*n* = 4)	*Physeter macrocephalus*, *Mesoplodon bidens*, *Ziphius cavirostris*, *Hyperoodon ampullatus*	281
**Harbour porpoises**	Harbour porpoise (*n* = 2676)	*Phocoena phocoena*	2676
**Pelagic dolphins**	Long-finned pilot whale (*n* = 275), white-beaked dolphin (*n* = 257), Risso’s dolphin (*n* = 204), striped dolphin (*n* = 138), Atlantic white-sided dolphin (*n* = 196), bottlenose dolphin (*n* = 119), killer whale (*n* = 28)	*Globicephala melas*,* Lagenorhynchus albirostris*,* Grampus griseus*,* Stenella coeruleoalba*,* Lagenorhynchus acutus*,* Tursiops truncates*,* Orcinus orca*	1217

### Annual trends

**Fig. 1:**
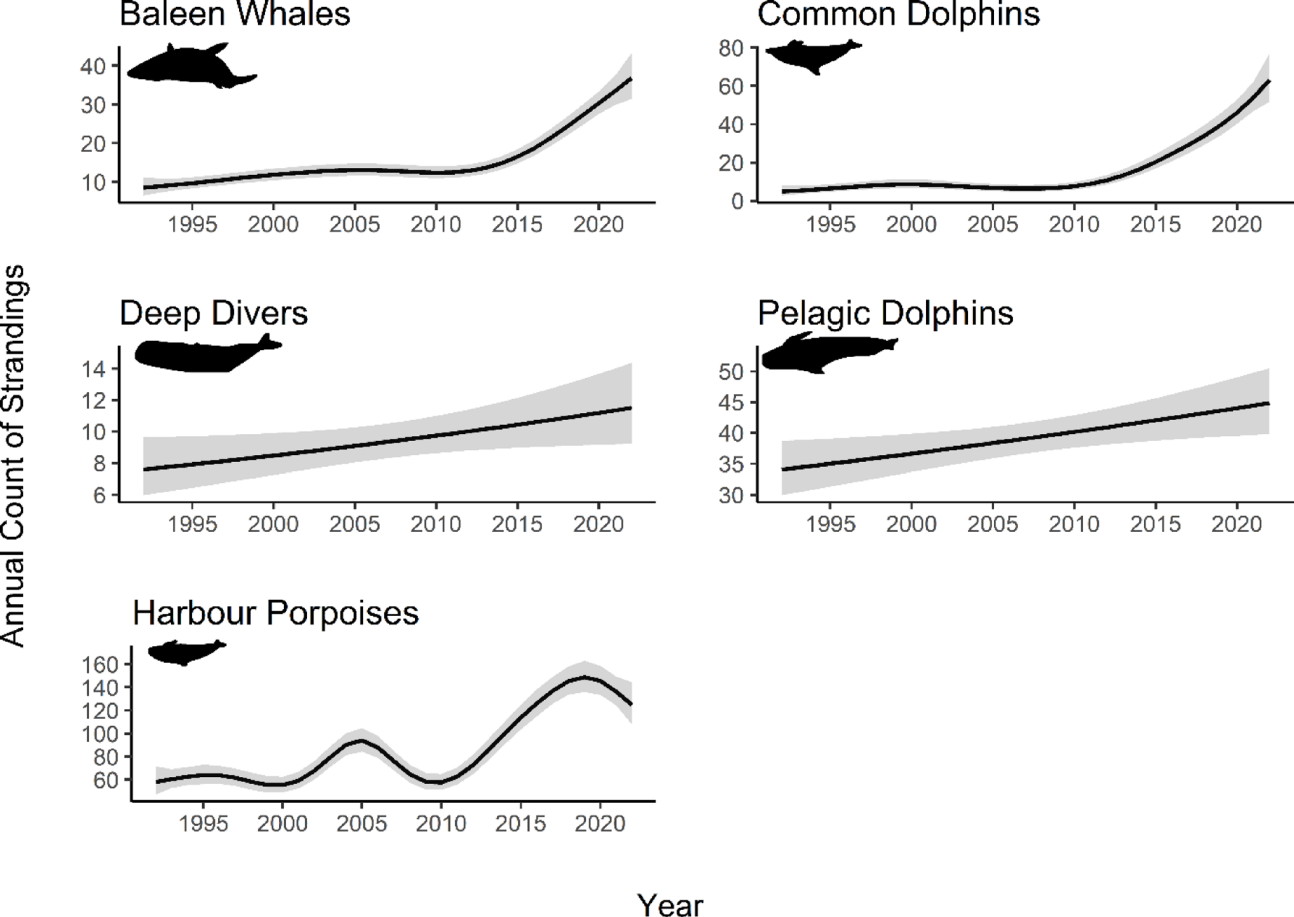
Trends in annual frequencies of single strandings shown in five species groupings. Black smoother curves generated from best fit GAMMs, with grey band representing 95% confidence intervals and grey points showing raw count data.

Each species group showed an increase in stranding numbers across the range of the scheme, with three distinct trend types (Fig. 1, Table S2). Baleen whales (edf = 2.991, *p* < 0.05) and common dolphins (edf = 4.95, *p* < 0.05) stranding rates were consistently low in the first two decades before increasing exponentially, with both showing a sharp increase around 2010. Deep divers (edf = 1.00, *p* > 0.05) and pelagic dolphins (edf = 1.00, *p* < 0.05) showed a linear increase, though this was non-significant for deep divers. Harbour porpoises had an oscillating pattern, but with an overall increase in strandings (edf = 7.46, *p* < 0.05). Sensitivity analysis showed these trends to hold across species for composite groups.

There were significant differences in annual stranding trends for age classes, with juveniles increasing more than other age classes in baleen whales (z = 4.27, *p* < 0.05), common dolphins (z = 4.92, *p* < 0.05) and deep divers (z = 2.76, *p* < 0.05). For pelagic dolphins, juvenile strandings increased more than adults (z = 2.17, *p* < 0.05) but the rate of increase was highest in neonates (z = 2.15, *p* < 0.05). Harbour porpoises showed no age class differences in annual stranding trends, with neonates, juveniles and adults increasing at an equal rate (all *p* > 0.05). There were no sex differences in annual stranding rates (baleen whales: z = -0.97, *p* > 0.05; common dolphins: z = 0.83, *p* > 0.05; deep divers z = -0.21, *p* > 0.05; harbour porpoises: z = 0.21, *p* > 0.05; pelagic dolphins: z = 0.48, *p* > 0.05).

### Seasonal trends

**Fig. 2:**
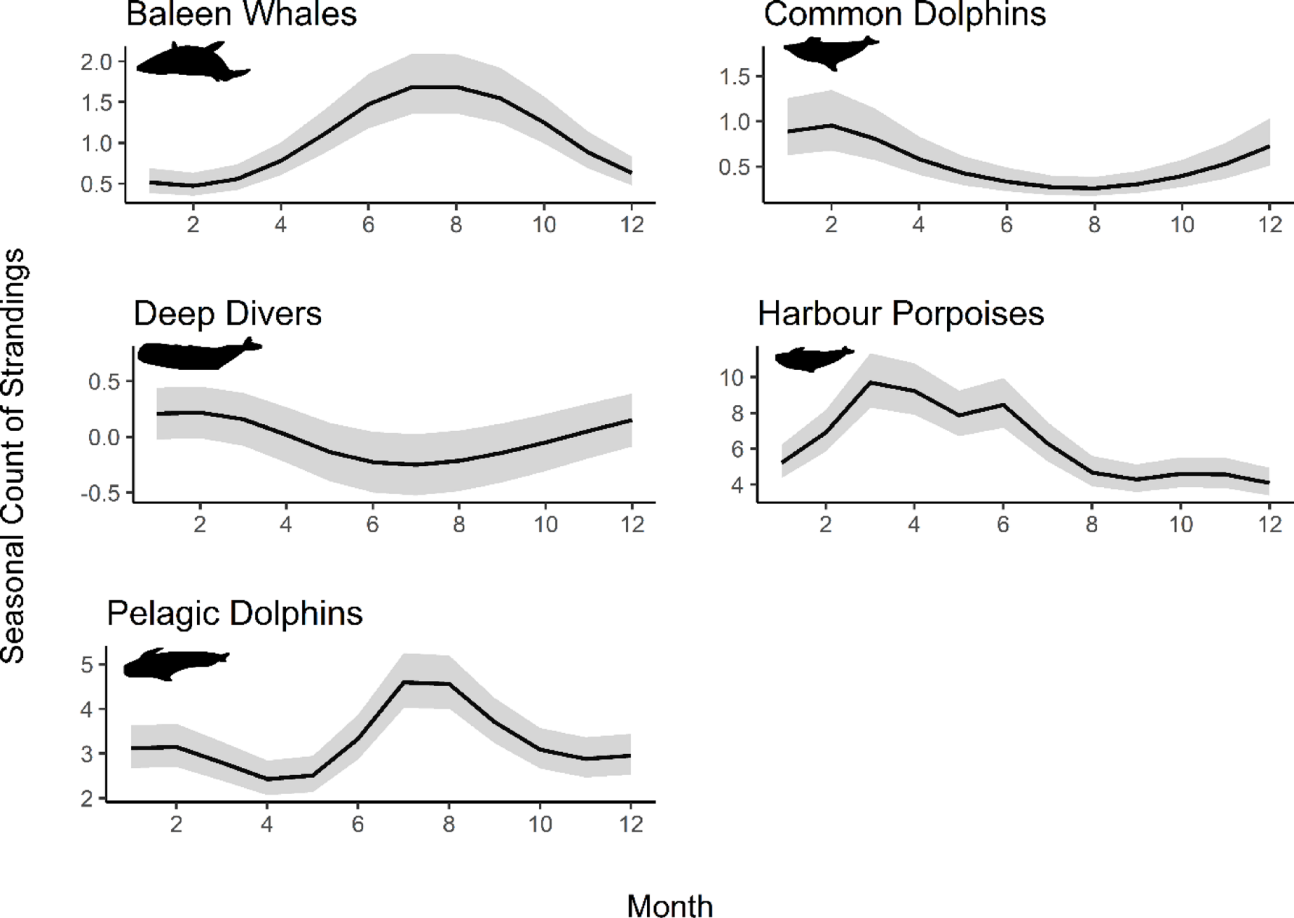
Trends in seasonal frequencies of single strandings shown in five cetacean species groupings with smoother curves generated from best fit GAMMs, and grey band representing 95% confidence intervals.

Distinct seasonal trends were found for each group (Fig. 2, Table S2). For baleen whales, monthly smoother curves showed a summer increase with a peak across July and August (edf = 3.97, *p* < 0.05). The same summer trend was found in pelagic dolphins (edf = 4.55, *p* < 0.05). For common dolphins, there was a winter increase with a peak in February (edf = 4.46, *p* < 0.05). For deep divers, there was an increase in both autumn and winter with a rise in strandings from October to February (edf = 1.79, *p* > 0.05). For harbour porpoises, there was an initial spring increase in March, followed by a second, smaller, summer increase in June (edf = 7.42, *p* < 0.05).

Sensitivity analysis revealed some differences in seasonal trends between species (Figure S1). For baleen whales, the seasonal trends are only significant in minke whales (edf = 1.35, *p* > 0.05), but not in humpback whales (edf = 0.00, *p* > 0.05) or in fin whales (edf = 0.00, *p* > 0.05). For deep divers, all species demonstrate the autumn and winter increase, but the precise peak varied, with Sowerby’s beaked whales peaking in October (edf = 1.55, *p* > 0.05), northern bottlenose whales in December (edf = 1.32, *p* > 0.05) and goose-beaked whales (edf = 2.21, *p* < 0.05) and sperm whales (edf = 0.96, *p* > 0.05) in January and February. For pelagic dolphins, bottlenose dolphins (edf = 2.80, *p* < 0.05), Risso’s dolphins (edf = 2.33, *p* < 0.05), striped dolphins (edf = 2.58, *p* < 0.05) and white-beaked dolphins (edf = 4.56, *p* < 0.05) all show a significant summer peak. Atlantic white-sided dolphins (edf = 0.00, *p* > 0.05), killer whales (edf = 0.00, *p* > 0.05) and long-finned pilot whales (edf = 0.00, *p* > 0.05) display a smaller, non-significant summer increase in strandings.

Amongst age classes, there were differences in stranding rates for each group (Figure S2, Table S3). The increase in summer strandings for baleen whales was seen in adults (edf = 3.71, *p* < 0.05) and juveniles (edf = 1.63, *p* < 0.05), but not neonates which displayed no seasonality (edf = 0.85, *p* > 0.05). For common dolphins, the increase in strandings in the winter was seen across age classes (A: edf = 3.20, *p* < 0.05, J: edf = 3.61, *p* < 0.05), N: edf = 1.22, *p* > 0.05). For harbour porpoises, adults showed no seasonality (edf = 0.53, *p* > 0.05), juveniles only peaked in March (edf = 5.21, *p* < 0.05) and neonates only in June (edf = 5.78, *p* < 0.05). In pelagic dolphins, the summer peak in strandings was seen across age classes (A: edf = 2.60, *p* < 0.05, N: edf = 3.28, *p* < 0.05) but juveniles had an additional peak in January (edf = 2.67, *p* < 0.05). Models of age class trends for deep divers did not converge due to small sample sizes.

There were no seasonal sex differences in baleen whales (M: edf = 2.72, *P* < 0.05, F: edf = 2.39, *p* < 0.05), common dolphins (M: edf = 2.93, *P* < 0.05, F: edf = 3.23, *p* < 0.05) or deep divers (M: edf = 1.44, *p* > 0.05, F: edf = 0.8, *p* > 0.05;). In harbour porpoises, more females than males stranded in March and June (edf = 6.89, *p* < 0.05), whereas the opposite was true in March, with more males than female harbour porpoises stranding (edf = 3.92, *p* < 0.05). For pelagic dolphins, more females than males stranded in July (edf = 3.56, *p* < 0.05) whereas in August more males than females stranded (edf = 3.91, *p* < 0.05).

### Spatiotemporal trends

**Fig. 3:**
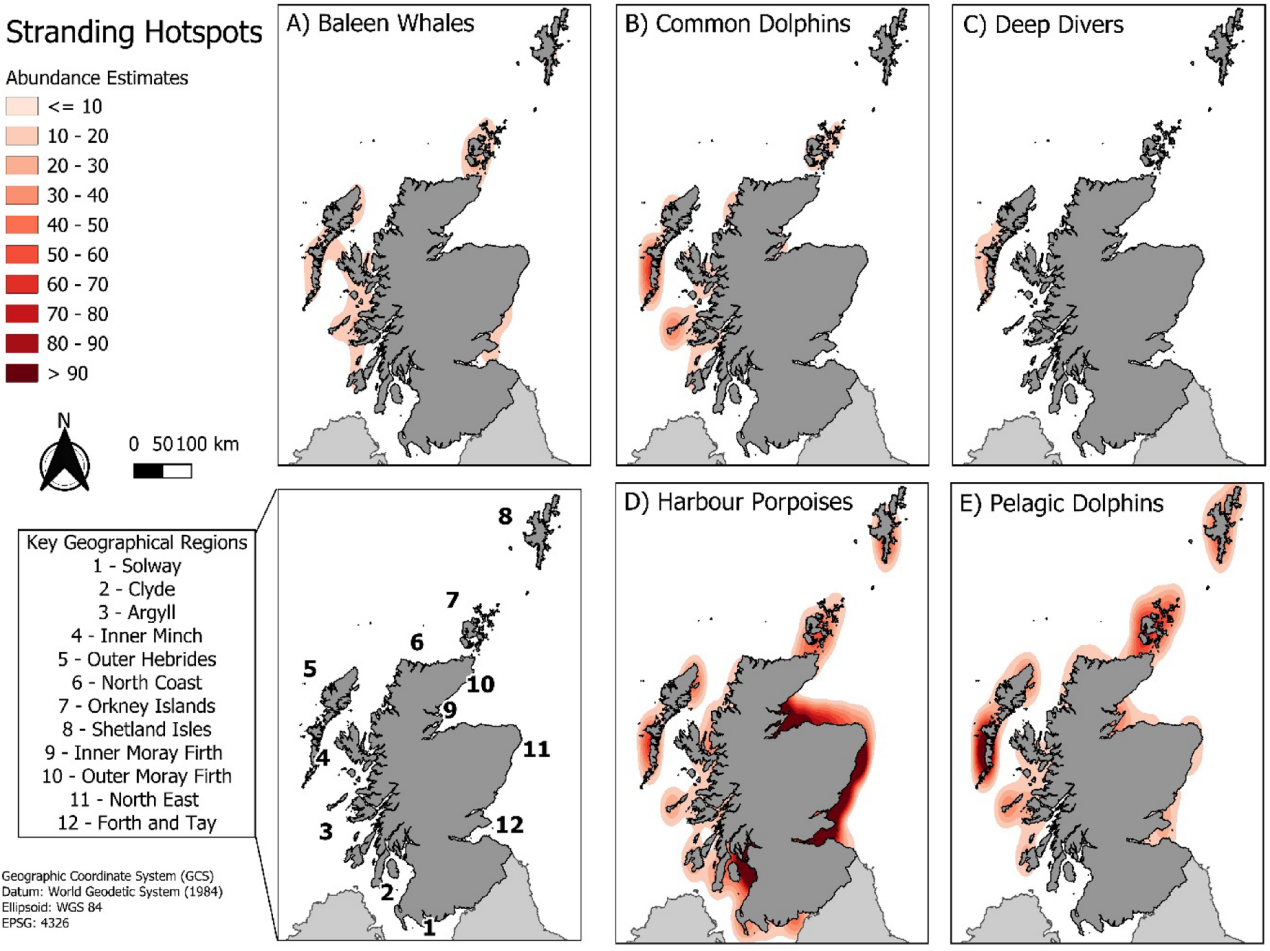
Spatial distribution of five cetacean species group’s stranding events in Scotland using a kernel density estimate with a 50 km radius, with darker reds showing a higher density of strandings. Key geographical regions highlighted in bottom left map.

Hotspot analysis of all cetacean stranding rates shows events widespread across Scotland, but with species specific spatial clustering (Fig. 3). Almost all species cluster to the north west coast, except porpoises, which predominantly cluster along the east coast around the Inner Moray Firth, the Outer Moray Firth and Forth and Tay, and the south east in the Clyde.

There was varied regional seasonality across species groups (Table 2, Table S4, reporting statistics in table S5). For baleen whales, summer increase in strandings were seen in all regions across Scotland except the North Coast, Orkney Islands and Solway which showed no seasonality. For common dolphins, winter peaks were exclusive to the west coast, namely the Clyde, Argyll, the Inner Minch, the Outer Hebrides and Orkney Island and Shetland Isles. For deep divers, most trends were non-significant but smoother plots showed winter increases in the north and northwest, namely Argyll, the Outer Hebrides, Orkney Islands, and Shetland Isles. An autumn increase was seen in the Southwest, in the Clyde and Solway. For harbour porpoises, there was a spatial distinction between the spring and summer peak, with the March peak occurring in the Forth and Tay, the Northeast as well as Orkney Islands, and Shetland Isles. The June peak for harbour porpoises was exclusive to the Inner Moray Firth, the North Coast, and the Outer Moray Firth. For pelagic dolphins, the summer peak is only seen in the Inner Minch, the North Coast, Orkney Islands, and the Inner Moray Firth.

**Table 2:** Seasonal Spatiotemporal trends in stranding rates for five cetacean species groups in Scotland.

	Baleen Whales	Common Dolphins	Deep Divers	Harbour Porpoises	Pelagic Dolphins
**Solway**	None	None	Autumn	None	None
**Clyde**	Autumn	Winter	Autumn	None	None
**Argyll**	Summer	Winter	Winter	None	None
**Inner Minch**	Summer	Winter	None	None	Summer
**Outer Hebrides**	Summer	Winter	Winter	None	None
**North Coast**	None	None	None	Summer	Summer
**Orkney Islands**	None	Winter	Winter	Spring	Summer
**Shetland Isles**	Summer	Winter	Winter	Spring	None
**Outer Moray Firth**	Summer	None	None	Summer	None
**Inner Moray Firth**	Summer	None	None	Summer	Summer
**Northeast**	Summer	None	None	Spring	None
**Forth and Tay**	Autumn	None	None	Spring	None

*Regions are listed in their geographical position, starting in the South West, and continuing clockwise to the South East.

## Discussion

Understanding population dynamics is a cornerstone of species monitoring, particularly for vulnerable populations facing multifactorial and increasing human and environmental pressures^19^. In this study, we used a 30-year dataset to identify key changes in spatiotemporal stranding patterns of five cetacean groups, demonstrating the valuable role of basic ‘Level A’ stranding data in identification of critical conservation opportunities. All groups showed rising stranding rates, with common dolphins and baleen whales identified as species of concern due to steep increases in stranding incidence. Distinct seasonal and regional trends also emerged, pointing to regions where enhanced monitoring and conservation efforts could be most effective. Stranding frequency changes may result from a mix of factors, ranging from shifts in cetacean distribution to variations in effort and mortality rates^22^. While determining the exact causes can be complex, identifying these general trends provides a practical starting point for targeting future research, underscoring the value of long-term stranding programs as monitoring tools able to guide conservation strategies.

We analysed annual trends to demonstrate how long-term data, and non-linear modelling can be used to identify species of concern and help prioritise further investigation, with trend types (linear, exponential, and oscillating) indicating the severity of the change and potential population impacts. Here, all cetacean species groups showed increased stranding rates over time. Populations with exponential trends in stranding rates should be prioritised for conservation efforts over those with linear (e.g., deep divers, pelagic dolphins) or oscillating (e.g., harbour porpoises) trends, as these groups represent slower increases which allow more time for interventions, marking them as lower priority. Baleen whales and common dolphins displayed exponential increases in stranding rates from approximately 2010. The increase was mostly characterised by juveniles for both groups, which may be due to successful recruitment in this age class from a growing population^46^. Alternatively, this may reflect a true increase in mortality rates, potentially from heightened exposure of younger, more vulnerable individuals to pressures, with possible population-level consequences if trends persist^4,7,8,17,36^. Further investigation could look into the cause of death of juveniles in these groups to elucidate if there is a specific cause of death driving the increase (i.e., entanglement, bycatch, infectious disease), or if there is a proportional rise across multiple causes, which would be more indicative of general population growth. This analysis helps to identify species groups of concern that require the most immediate conservation attention, prioritising future investigation and ensuring resources are effectively allocated.

We also showed that harbour porpoises exhibit an oscillating trend in stranding rates, with three peaks per decade. The magnitude of these peaks has increased over time, but the relatively short time period between fluctuations (~ 5 years) suggests these are unlikely to be driven by mortality or sustained anthropogenic pressure. Instead, the pattern may represent inter-annual variation in population abundance, as other studies have shown small scale migrations in harbour porpoise in response to oceanographic conditions, such as sea surface temperature^37–39^. It is possible that porpoises are moving between Scotland, the rest of the UK and even further across the North Sea. As this study only observes their presence in Scotland, it does not capture wider, international trends in movements. Ecosystem-scale integration and harmonisation across stranding networks would help to clarify these patterns and better characterise the large-scale movements of mobile species.

Using broad-scale spatiotemporal analysis, we demonstrate how distinct spatial clustering and seasonal patterns provides valuable insight on species trends on a scale that is both relevant to the transient species being monitored, and maintains legislative boundaries for downstream, targeted conservation approaches. Here, analysis revealed distinct seasonality for each cetacean group with specific spatial clustering. Most stranding groups clustered to the west coast, except for porpoises, which predominantly stranded on the east. Clustering of strandings may indicate seasonal population aggregations, such as specific mating sites or migratory routes, marking these as high-risk areas for that species. For example, baleen whales are thought to use the west coast of Scotland as a resting point during seasonal migrations between high latitude feeding grounds and low latitude mating grounds, which may explain why strandings cluster to this coastline^46^. Common dolphins also cluster to the west coast but display a winter specific increase in strandings, which is different from the summer trend observed in pelagic dolphins. Similar winter increases have been reported elsewhere in the northeast Atlantic and are often attributed to bycatch, which may also explain the pattern observed here^40^. The combination of this seasonal spike and exponential increases in annual strandings highlights common dolphins as a priority species for further investigation into the potential role of fisheries interactions in this region.

Spatiotemporal analysis also revealed distinct seasonal and regional stranding patterns in harbour porpoises, offering insights into key life history stages and potential periods of increased vulnerability. Harbour porpoise strandings increased in the spring and early summer, matching trends seen in Belgium, the Netherlands, and Germany^41–43^. In this study, the initial peak in March mainly consisted of juveniles stranding in the south-east of Scotland, characterised by high first-year mortality rates of recently weaned animals^41^. The second peak in June was dominated by neonates in the Inner and Outer Moray Firth, coinciding with summer calving season in Scotland, suggesting this may be an important region for reproduction in this species and an important area for implementing protective measures^44^. Identifying the regions and times where cohorts of animals may be least resilient is important as it can indicate when additional anthropogenic pressures could have the most significant impact. For example, preventing natural calf mortality cannot be easily achieved, but reducing additional anthropogenic pressures, such as disturbance from underwater noise and vessel activity, on juveniles in periods when additional stressors might critically diminish their resilience is most likely to be successful in preventing excess mortalities^22^. Furthermore, the Moray Firth SAC is a known site of aggressive bottlenose dolphin interactions which may be contributing to the rise in strandings observed in this area, particularly if harbour porpoises are more abundant in this region during the calving season, resulting in increased number of interactions^45^.This represents a valuable area for future investigation, which could incorporate post-mortem examinations and behavioural data within this hotspot to determine drivers of observed seasonal mortality. Understanding demographic trends across species group will inform the most appropriate conservation strategies, and the locations and periods they would be most successful.

Determining the drivers of observed changes can be difficult, but there are several key factors that are important to consider. The variation in stranding rates seen may be driven by changes in population abundance and distribution causing a proportional rise in stranding rates. Here, baleen whale and pelagic dolphin strandings peaked in the summer, which is also when they are typically present in Scottish waters, according to sightings data^46,47^. Higher numbers of baleen whales have been observed in northern latitudes in recent years, suggesting increasing stranding incidence may be related to higher abundance^47^. Similarly, reports have demonstrated a south to north range shift in common dolphins and striped dolphins over the past three decades which is thought to be partly caused by movements in prey^17,48^. The results of this study may also suggest a similar range shift in deep divers, as historically only adult male sperm whales stranded in the UK, reflecting the segregation between females with their young, and bulls^49,50^. The significant rise in juvenile sperm whale stranding rates found here suggests a potential shift in habitat use, with younger individuals being driven northwards, potentially in response to climate-induced shifts in target prey^51–53^. Further work could, for example, utilise species distribution overlays or long-term sightings data to assess whether observed trends reflect broader changes in the movements of deep diver species through their northern ranges. Identifying potential range shifts through stranding data can provide a foundation for further investigation into climate change impacts on marine ecosystems and wildlife.

Human activities are steadily intensifying within ocean ecosystems, likely impacting wildlife populations^1^. Noise pollution is a critical stressor for marine mammals, especially for deep-diving species^55–57^. In Scotland, sources of anthropogenic noise are abundant, including seismic survey air guns and drilling from industrial construction, both of which are expected to increase due to offshore wind farm projects aimed at achieving 2050 energy targets^58,59^. Monitoring cetacean populations prior to, during and post development is imperative to understand the impacts of these industrial activities. For baleen whales, entanglement remains a significant threat, exacerbated by increased fishing activity in parallel with growing whale populations in Scottish waters as they rebound from the cessation of whaling^8,54^. Studies suggest that juveniles are particularly susceptible to entanglement, which may help explain the age-related patterns observed in this study^36^. The present study does not include cause of death data, but this information is routinely collected on a subset of cases through necropsy, supported by ancillary diagnostic tests and advanced supplementary tools (e.g., virtopsy) and can be harnessed to provide insight into the specific threats affecting individuals^29,60,61^. While this deeper level of data is extremely valuable and should be collected wherever possible to elucidate and quantify likely drivers of mortality, and possible evidence of cumulative, sub-lethal impacts, such approaches are often limited by logistical, financial, and practical challenges. Initial spatiotemporal analysis as outlined here provides a valuable foundation for guiding and prioritising further investigations, helping to identify the species and regions where enhanced data collection is likely to be most impactful. For example, the observed trends in juvenile baleen whale mortality could warrant targeted assessment of the potential role of entanglement in this group. By focusing on the most at-risk populations and those with rising stranding rates, strandings investigation teams can better target resources to best address the conservation needs of vulnerable species.

It is also relevant to consider the impacts of sampling effort and how this may be influencing temporal trends. Throughout the time period analysed in this study, accompanying metadata on recorder effort was not collected in conjunction with stranding data. Although this represents a limitation of the dataset, this is not unusual with strandings data and there is currently no agreed method between strandings networks as to how effort metrics can best be assessed. The awareness and use of stranding schemes has grown in conjunction with the rise of social media and enhanced internet connectivity which enables rapid dissemination of information and aids ease of reporting^62,63^. This increased visibility in recent years can lead to more frequent reports of stranded animals, making it difficult to distinguish between genuine ecological changes and increases in reporter effort^64^. In this study, increases in stranding rates were observed across all cetacean species groups. However, varying trend types (e.g., linear, exponential, or oscillating trends, Fig. 1) were seen across species groups which indicates a true biological cause underlying the observed patterns as trend effects would be similar across species groups if they were solely driven by reporter effort. Similarly, regional hotspots varied between species groups, suggesting genuine biological patterns rather than effort artifacts, since reporter effort should affect all species equally. Therefore, it can be assumed that true ecological changes can be elucidated using the approach described here. However, it is recommended that stranding schemes endeavour to identify and employ a measure of reporting frequency to allow for more robust effort-based corrections going forward.

This study highlights the value of using long-term, opportunistically collected ‘Level A’ data for monitoring species population trends. Although the resolution of data varies across stranding programs, nearly all schemes gather baseline, ‘Level A’ spatiotemporal data. The use of long-term datasets and adaptable models such as GAMs, is particularly valuable for capturing complex, generational changes in long-lived species. For example, the over-20-year span required to reveal annual oscillations in harbour porpoises demonstrates that shorter datasets might only provide misleading snapshots. The effectiveness of this method also suggests potential applications in other wildlife monitoring programs based on opportunistic public or volunteer surveillance, underscoring the role of public engagement in collecting valuable data and supporting timely, data-driven conservation strategies^65^.

Here, we have demonstrated that ‘Level A’ stranding data can be used to establish population trends, prioritise at-risk species and target conservation approaches for cryptic species. Our annual trend analysis of five cetacean groups identified increasing trends in stranding rates throughout Scotland. We, therefore, recommend that research efforts on baleen whales and common dolphins should be prioritised to elucidate the driver of observed exponential increases in stranding rates. In addition, we demonstrated that spatiotemporal trends can be used to target conservation strategies to ensure efficient use of time and resources and maximise chances of successful outcomes. Despite the challenges of using opportunistic data, the insights gained are invaluable and not easily obtained through other means. This demonstrates the value of baseline stranding data as a monitoring tool for cetaceans, even when collected opportunistically. Standardising this approach across stranding programmes will enhance our ability to detect population trends and develop appropriate conservation approaches for these cryptic marine species.

## Methods

### Data collection

Data were obtained from the Scottish Marine Animal Stranding Scheme (SMASS), where strandings are reported opportunistically by the public or trained volunteers and validated by an experienced stranding coordinator. For each case, ‘Level A’ data were collected, which is comprised of species, date, location of stranding, and sex and age class of the individual (Figure S3). Sex was determined by visual assessment, and age class was determined by body length^66–68^. Depending on the condition of the animal, further information may be collected, but for the purpose of this study, only ‘Level A’ data were used^28^. It is relevant to note that recorder effort was not accounted for in this analysis, as no effort-related meta-data were collected during the original data acquisition. Data from the commencement of the stranding scheme on 1st January 1992 until 31st December 2022 were included.

Only single stranding events were used in this study, with Unusual Mortality Events (significant die-offs of marine animal populations) and Mass Stranding Events (events that involve more than two individuals that are not mother-calf pairs) removed to avoid skewing trends^69^. For the deep diver’s group, this meant removing events from 2018 in its entirety due to an unusual mortality event that year that skewed baseline findings in initial data exploration^70^.

### Species grouping and dataset generation

There were 17 cetacean species included in this study, with very rare species (< 5 total recordings) removed from the datasets used for the purposes of analysis. The remaining species were separated into five distinct stranding groups based on broadly shared ecological similarities: baleen whales, short-beaked common dolphins, deep divers, harbour porpoises and pelagic dolphins (Table 1). Short-beaked common dolphins were grouped separately due to initial data exploration indicating seasonal trends that deviated from other pelagic dolphins, as well as overall higher stranding rates. Long-finned pilot (*Globicephala melas*) whales were grouped with pelagic dolphins as, despite their capacity to dive to significant depths, their habitat use, and behaviour are more similar to that of other pelagic dolphins^71^. Only cases with confirmed species identification were included in each group. However, any carcase identified as an indeterminate baleen whale or indeterminate beaked whale species were also included in their respective group.

Datasets of stranding counts of single strandings per month and year were generated for each of the five groups. For demographic analysis, datasets of counts per month were generated with region, sex and age variables included.

### Spatial regions

**Fig. 4:**
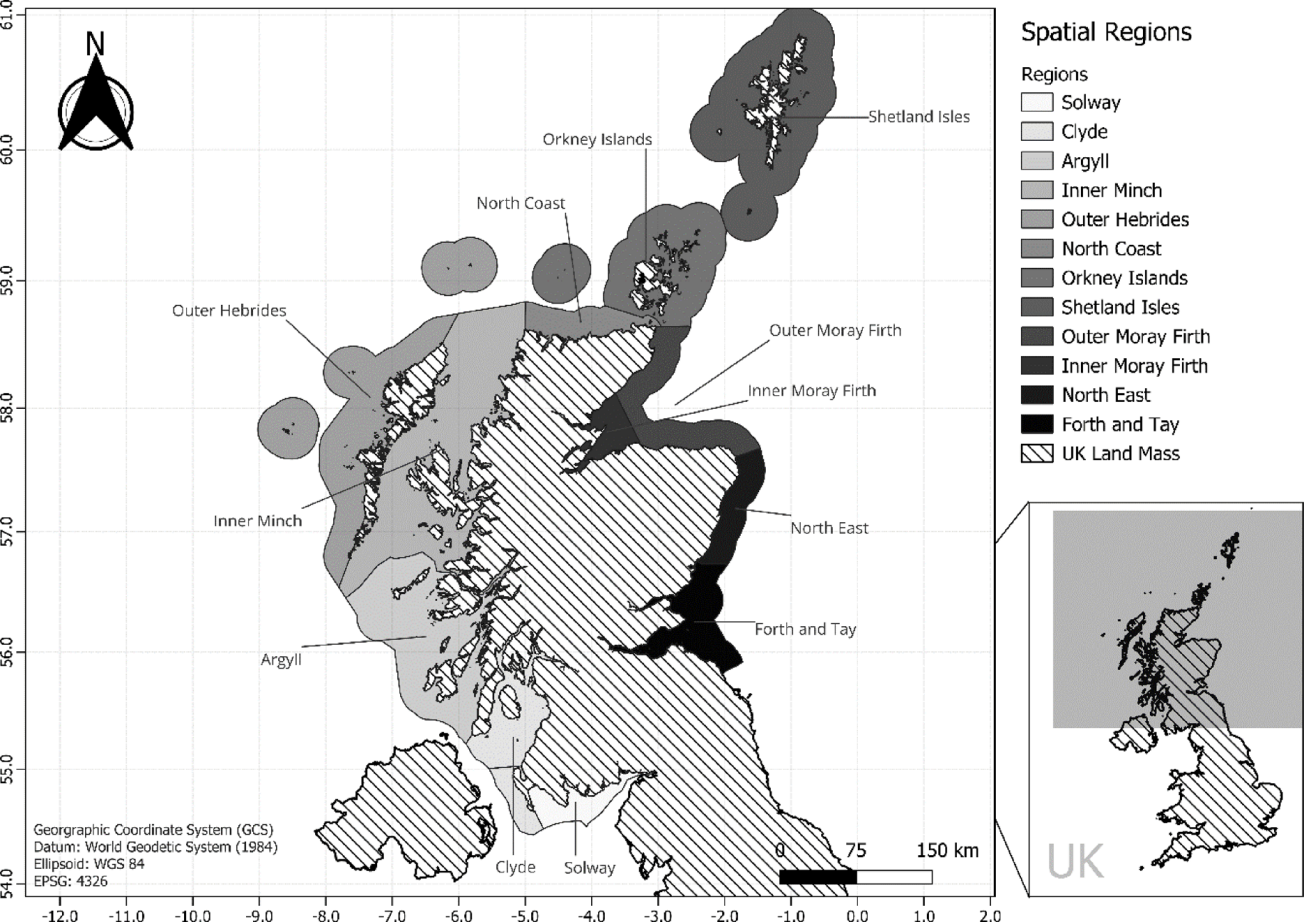
Map of Scotland and 12 associated spatial regions used for spatial analysis of cetacean strandings. Adapted from the Scottish Marine Regions to include the Special Area of Conservation of the Inner Minch, and the Inner Moray Firth^67^.

A total of 12 regions were established for spatial analysis of stranding rates (Fig. 4), derived from Scottish Marine Regions (SMR), and adapted to include the Special Area of Conservation (SAC) of the Inner Minch, and the Inner Moray Firth^72^. The stranding process is subject to various environmental influences including tide, wind, and currents. Thus, these large regions encompass the area in which each animal has the potential to strand, as well as where it may have lived immediately prior to stranding^25^. This level of resolution provides insight into spatial trends whilst still allowing for natural variation in the stranding process and simultaneously maintaining legislative boundaries for downstream monitoring applications.

### Temporal analyses

All data analysis was carried out using the statistical software programme R (version 4.2.2)^73^. A table of all models used can be found in the supplementary materials (Table S1). Extensive data exploration was conducted prior to analysis to test for collinearity, zero inflation and to inform model choice.

To analyse seasonal and annual trends in stranding rates, a Generalised Additive Mixed Model (GAMM) was fit to count datasets using the *mgcv* package^74^. Data exploration revealed non-linear trends in the data indicating that this would be the most appropriate model choice as the flexibility of these models allows non-linearity to be captured. A smooth term of month was fit to the model with a cyclic cubic spline to account for the cyclical nature of months. The knots of this spline, which indicate its piecewise boundaries, were set to 12 at 0.5–12.5 with an interval of 1 to allow January and December to be equidistant to other consecutive months. A second smoother of year with a thin plate regression spline was also fit to the model. Various iterations of the model were tested, with inclusion of interaction terms of year and month and with year as a random effect. Models were fit to either a Poisson error distribution and log-link function or a negative binomial distribution depending on the dispersion of the data, tested with residual deviance to residual degrees of freedom ratios. Data distribution was assessed and if there was a high number of zeroes, zero inflated Poisson (ziP) structures were fit to a GAM and compared to models with other distributions.

Due to the collinearity of the timeseries data, an autoregressive moving average process (ARMA) with a first order correlation structure was also tested in the models with the ARMA nested within year to account for continuous autocorrelation. The autocorrelation factor (ACF) and partial autocorrelation factor (pACF) plots of normalised residuals were assessed for remaining autocorrelation. Final models were selected based on assessment of goodness of fit through visual interpretation of diagnostic plots and comparison of Akaike Information Criterion (AIC) values.

For all final models, sensitivity checks were carried out by removing random months or years and adding pseudo-years of randomly generated counts to ascertain if trends change with small adjustments. All models were robust to these changes. For groups with more than one species, random species were added and removed to ensure that there were no masked trends. Some groups, such as the pelagic dolphins, showed variation between species and so additional models with species type as a covariate were fit to extract these unique trends. Figures were then generated using the *ggpredict* and *ggplot* functions from the ggplot2 package^75^.

### Demographic trends

To test for demographic trends, generalised linear models (GLMs) were fit to sex and age group with an interaction of year to assess differences within the levels of the variables and determine annual trends. Seasonal differences in demographics were analysed with GAMM models fit to a smooth of month and interaction of the demographic covariates and a smooth of year. Models with year as a random intercept, and with and without ARMA models were also tested. Final models were selected by comparing AIC values, and goodness of fit was confirmed through visual interpretation of diagnostic plots.

### Spatial analyses

Spatial analysis was carried out to determine clustering of strandings across Scotland. Stranding events were analysed as point data using the latitude and longitudes of each stranded animal in WGS EPSG:4326 projection. A heatmap was generated using a kernel density estimation in QGIS across 5, 50, 100, 150 and 200 km search radius with 50 km ultimately chosen as the most appropriate distance based on visual interpretation of raster outputs^76^. Heatmaps were then created to display aggregations of stranding events, with step sizes of 10 to allow comparative interpretation across species groups.

### Spatiotemporal analyses

A regional shapefile was generated in QGIS using the *split features* tool to modify the SMR shapefile and SAC shapefile to include all 12 spatial regions (Fig. 4)^76^. The region shapefile was then imported to R and the *nearest polygon* tool in the *spatstat* package was used to join data points to relevant regions^76,77^. Spatial autocorrelation between regions based on stranding rates for each species were tested using Moran’s I Monte Carlo test with nearest neighbour analysis. No spatial autocorrelation was found for any of the species’ groups across regions and thus no spatial autocorrelation structure was used in the final models.

To test for regional seasonality, GAM models were fit to regional count datasets following the methodology described under the temporal analysis subsection, but with an additional region variable. A fixed effect of region was tested in models with a Markov Random Field (MRF) smooth to allow for modelling of the connections between regions based on a nearest neighbour matrix. Model distribution and best fit was established in the same way as described previously for temporal models.

For all final spatial models, sensitivity checks were carried out by changing the boundaries of each region by 1, 5 and 10 km and rerunning models to determine if trends changed based on these small adjustments. For all species groups, the 1 km and 5 km shifts resulted in no change in the significance of each region or the trends of smoother curves. For the baleen whales and the pelagic dolphins, the 10 km boundary shift resulted in two regions no longer being significant but the original trend could still be seen in smoother plots. Therefore, it can be concluded that the boundaries chosen for each region were robust.

## Electronic supplementary material

Below is the link to the electronic supplementary material.

Supplementary Material 1

## Data Availability

The datasets generated during and analysed during the current study are available from the corresponding author on reasonable request.

## Notes

**Tables**.s1
