# Supplementary material for: An approach to using stranding data to monitor cetacean population trends and guide conservation strategies
Source: Sci Rep. 2025 Aug 20;15:28417. doi: 10.1038/s41598-025-12928-1 (PMC12368117; doi:10.1038/s41598-025-12928-1)
Supplement: Supplementary file 1 — Supplementary Material 1 [file 41598_2025_12928_MOESM1_ESM.docx]

***An Approach to Using Stranding Data to Monitor Cetacean Population Trends and Guide Conservation Strategies***

Rachel L. Lennon^a, b*^, Rosie S. Williams^c^, Kathryn J. Allan^a^, Mariel T.I. ten Doeschate^a^, Nicholas J. Davison^a^, Simon A. Babayan^a^, Andrew C. Brownlow^a^

^a^ School of Biodiversity, One Health and Veterinary Medicine, University of Glasgow, Graham Kerr Building, University Avenue, Glasgow, G12 8QQ, UK

^b^ Centre for Ecology and Conservation, University of Exeter, Penryn Campus, Penryn, Cornwall TR10 9EZ, UK

^c^ Zoological Society of London, Institute of Zoology, Regent’s Park, London NW1 4RY, UK

*Corresponding author: Rachel Lennon ([r.lennon.3@research.gla.ac.uk](mailto:r.lennon.3@research.gla.ac.uk))

**Supplementary Materials**

Supplementary Tables

Table S1. A list of all models and associated variables used for analysis of spatiotemporal trends in cetacean strandings in Scotland.

| Purpose of model | Model type | Variables | Distribution | Additional structures |
| --- | --- | --- | --- | --- |
| Seasonal & annual trends | Generalised Additive Mixed Model | s(month, bs = “cc), s(year) | Poisson, negative binomial or zero inflated Poisson | ARMA correlation structure |
| Spatial trends | Generalised Additive mixed model | s(month, bs = “cc”), s(year), s(region, bs = “mrf”) | Poisson, negative binomial | ARMA correlation structure |
| Annual demographic trends | Generalised linear model | year*age, year*sex | Poisson, negative binomial | NA |
| Seasonal demographic trends | Generalised Additive mixed model | s(month, bs = “cc, by = demographic), s(year, bs = “re”), deomgraphic | Poisson, negative binomial | ARMA correlation structure |
| Spatial demographic trends | Generalised linear model | region*demographic | Poisson, negative binomial | NA |

Table S2: Various combinations and AIC values for the temporal models that were compared to assess for temporal patterns in stranded animals. For each model we report the variables where s is the variable is fitted as a smooth, re as a random effect and * as an interaction. We include the Akaike Information Criterion (AIC), the value relative to the model with the lowest AIC (ΔAIC), the adjusted R^2^ value (note these are estimates for GAMs), and the phi value where applicable. The chosen model is shown in bold.

| species | year | month | ARMA | AIC | ΔAIC | R^2^adj | Phi |
| --- | --- | --- | --- | --- | --- | --- | --- |
| Baleen whales | s | s | - | 1032.76 | 0.00 | 0.38 | NA |
|  | s | re | - | 1043.08 | 10.32 | 0.31 | NA |
|  | s | * | - | 1099.22 | 66.46 | 0.41 | NA |
|  | **s** | **s** | **+** | **1080.24** | **47.47** | **0.37** | **-0.06** |
|  | s | re | + | 1122.85 | 90.09 | 0.14 | 0.14 |
| Common dolphins | s | s | - | 989.78 | 0.00 | 0.37 | NA |
|  | s | re | - | 1044.63 | 54.85 | 0.31 | NA |
|  | s | * | - | 1002.411 | 12.63 | 0.41 | NA |
|  | **s** | **s** | **+** | **1243.64** | **253.86** | **0.51** | **0.09** |
|  | s | re | + | 1456.63 | 466.85 | 0.09 | 0.48 |
| Deep divers | s | s | - | 905.39 | 19.24 | 0.02 | NA |
|  | s | re | - | 905.22 | 19.07 | 0.02 | NA |
|  | s | * | - | 886.15 | 0.00 | 0.20 | NA |
|  | s | s | + | 1309.39 | 423.24 | 0.02 | 0.07 |
|  | s | re | + | 1310.27 | 424.12 | 0.02 | 0.08 |
|  | **s** | **s** | **(ziP)** | **895.10** | **8.95** | **Deviance explained: 3.86%** | **NA** |
| Pelagic dolphins | s | s | - | 1601.17 | 794.78 | 0.11 | NA |
|  | s | re | - | 1607.56 | 801.17 | 0.09 | NA |
|  | s | * | - | 1607.98 | 801.59 | 0.25 | NA |
|  | **s** | **s** | **+** | **806.39** | **0.00** | **0.11** | **0.08** |
|  | s | re | + | 814.00 | 7.61 | 0.09 | 0.10 |
| Harbour porpoise | s | s | - | 2036.62 | 1327.57 | 0.39 | NA |
|  | s | re | - | 2125.81 | 1416.76 | 0.33 | NA |
|  | s | * | - | 1891.83 | 1182.78 | 0.50 | NA |
|  | **s** | **s** | **+** | **709.05** | **0.00** | **0.40** | **0.21** |
|  | s | re | + | 906.75 | 197.70 | 0.17 | 0.34 |

Table S3: Various combinations and AIC values for the demographic models that were compared to assess for demographic patterns in temporal trends of stranded animals. For each model we report the variables where s is the variable fitted as a smooth, re as a random effect and * as an interaction. We include the Akaike Information Criterion (AIC), the value relative to the model with the lowest AIC (ΔAIC), the adjusted R^2^ value (note these are estimates for GAMs), and the Phi value were applicable. The chosen model is shown in bold.

| species | year | month | sex | age | ARMA | AIC | ΔAIC | R^2^adj | Phi |
| --- | --- | --- | --- | --- | --- | --- | --- | --- | --- |
| Baleen whales | s | s | * | - | - | 870.68 | 0.00 | 0.09 | NA |
|  | s | re | * | - | - | 877.38 | 6.70 | 0.06 | NA |
|  | **s** | **s** | ***** | **-** | **+** | **3352.09** | **2481.41** | **0.08** | **-0.10** |
|  | **s** | **s** | **-** | ***** | **-** | 900.16 | 0.00 | 0.26 | NA |
|  | s | re | - | * | - | 926.89 | 26.73 | 0.20 | NA |
|  | **s** | **s** | **-** | ***** | **+** | **6159.46** | **5259.30** | **0.25** | **0.05** |
| Common dolphins | s | s | * |  | - | 979.02 | 0.00 | 0.27 | NA |
|  | s | re | * |  | - | 1010.52 | 31.50 | 0.22 | NA |
|  | **s** | **s** | ***** |  | **+** | **3380.28** | **2401.26** | **0.27** | **-0.03** |
|  | **s** | **s** |  | ***** | **-** | 1125.82 | 0.00 | 0.47 | NA |
|  | s | re |  | * | - | 1208.45 | 82.63 | 0.39 | NA |
|  | **s** | **s** |  | ***** | **+** | **6781.68** | **5655.86** | **0.47** | **0.00** |
| Deep divers | s | s | * |  | - | 822.27 | 2.68 | 0.06 | NA |
|  | s | re | * |  | - | 819.59 | 0.00 | 0.06 | NA |
|  | **s** | **s** | ***** |  | **+** | **3439.47** | **2619.88** | **0.05** | **0.11** |
|  | **s** | **s** |  | ***** | **-** | 863.65 | 0.00 | 0.08 | NA |
|  | s | re |  | * | - | 879.43 | 15.78 | 0.07 | NA |
|  | **s** | **s** |  | ***** | **+** | **7585.08** | **6721.43** | **0.09** | **0.05** |
| Pelagic dolphins | s | s | * |  | - | 1843.33 | 0.00 | 0.09 | NA |
|  | s | re | * |  | - | 1856.58 | 13.25 | 0.05 | NA |
|  | **s** | **s** | ***** |  | **+** | **2347.74** | **504.41** | **0.08** | **-0.03** |
|  | s | s |  | * | - | 2229.11 | 0.00 | 0.24 | NA |
|  | s | re |  | * | - | 2259.99 | 30.88 | 0.21 | NA |
|  | **s** | **s** |  | ***** | **+** | **4400.72** | **2171.61** | **0.23** | **0.03** |
| Harbour porpoise | s | s | * |  | - | 2610.47 | 639.65 | 0.21 | NA |
|  | s | re | * |  | - | 2662.52 | 691.70 | 0.16 | NA |
|  | s | s | * |  | + | 1970.82 | 0.00 | **0.21** | **0.06** |
|  | **s** | **s** |  | ***** | **-** | **2982.92** | 0.00 | 0.45 | NA |
|  | s | re |  | * | - | 3096.80 | 113.88 | 0.39 | NA |
|  | s | s |  | * | + | 4059.37 | 1076.45 | **0.45** | **0.03** |

Table S4: Various combinations and AIC values for the spatiotemporal models that were compared to assess for spatial patterns in temporal trends of stranded animals. For each model we report the variables where s is the variable fitted as a smooth, and re as a random effect. We include the Akaike Information Criterion (AIC), the value relative to the model with the lowest AIC (ΔAIC), and the adjusted R^2^ value (note these are estimates for GAMs). The chosen model is shown in bold.

| species | year | Month | region | AIC | ΔAIC | R^2^adj |
| --- | --- | --- | --- | --- | --- | --- |
| Baleen whales | **s** | **s** | **+** | **2798.31** | **0.00** | **0.12** |
|  | s | re | + | 2808.46 | 10.15 | 0.11 |
|  | - | s | + | 2877.65 | 79.34 | 0.08 |
| Common dolphins | **s** | **s** | **+** | **2498.62** | **0.00** | **0.29** |
|  | s | re | + | 2552.64 | 54.02 | 0.26 |
|  | - | s | + | 2849.44 | 350.82 | 0.10 |
| Deep divers | **s** | **s** | **+** | **1935.29** | **0.00** | **Deviance explained: 64%** |
|  | s | re | + | 1936.06 | 0.77 | Deviance explained: 64.5% |
|  | - | s | + | 1936.07 | 0.78 | Deviance explained: 64.5% |
| Pelagic dolphins | **s** | **s** | **+** | **5371.46** | **0.28** | **0.12** |
|  | s | re | + | 5371.28 | 0.10 | 0.12 |
|  | s | - | + | 5371.18 | 0.00 | 0.12 |
| Harbour porpoise | s | s | + | **5804.18** | 0.00 | **0.28** |
|  | s | re | + | 8592.85 | 2788.67 | 0.27 |
|  | s | - | + | 8829.16 | 3024.98 | 0.21 |

Table S5: Spatiotemporal trends and associated statistical significance of five cetacean species groups stranding rates in Scotland.

|  | **Baleen Whales** | **Common Dolphins** | **Deep Divers** | **Harbour Porpoises** | **Pelagic Dolphins** |
| --- | --- | --- | --- | --- | --- |
| **Solway** | None edf = 0.00, p > 0.05) | None edf = 0.0, p > 0.05 | Autumn, edf = 2.09, p > 0.05 | None edf = 0.0, p > 0.05 | None edf = 0.0, p > 0.05 |
| **Clyde** | Autumn edf = 3.35, p < 0.05 | Winter edf = 2.39, p < 0.05 | Autumn edf = 2.98, p < 0.05 | None edf = 0.0, p > 0.05 | None edf = 0.0, p > 0.05 |
| **Argyll** | Summer edf = 2.25, p < 0.05 | Winter edf = 3.56, p < 0.05 | Winter edf = 1.81, p < 0.05 | None edf = 0.0, p > 0.05 | None edf = 0.0, p > 0.05 |
| **Inner Minch** | Summer edf = 2.82, p < 0.05 | Winter edf = 3.55, p < 0.05 | None, edf = 0.82, p > 0.05 | None edf = 0.0, p > 0.05 | Summer edf = 1.77, p < 0.05 |
| **Outer Hebrides** | Summer edf = 2.34, p < 0.05 | Winter edf < 2.53, p < 0.05 | Winter edf = 1.89, p < 0.05 | None edf = 0.0, p > 0.05 | None edf = 0.0, p > 0.05 |
| **North Coast** | None edf = 0.08, p > 0.05 | None edf = 0.0, p > 0.05 | None edf = 0.0, p > 0.05 | Summer edf = 2.26, p < 0.05 | Summer, edf = 2.22, p < 0.05 |
| **Orkney Islands** | None edf = 0.22, p > 0.05 | Winter edf = 2.39, p < 0.05 | Winter, edf = 1.50, p > 0.05 | Spring edf = 2.07, p < 0.05 | Summer, edf = 2.74, p < 0.05 |
| **Shetland Isles** | Summer  edf = 2.87, p < 0.05 | Winter edf – 1.91, p < 0.05 | Winter, edf = 1.153, p > 0.05 | Spring edf = 2.24, p < 0.05 | None edf = 0.0, p > 0.05 |
| **Outer Moray Firth** | Summer edf = 1.92, p < 0.05 | None edf = 0.0, p > 0.05 | None edf = 0.0, p > 0.05 | Summer edf = 3.89, p < 0.05 | None edf = 0.0, p > 0.05 |
| **Inner Moray Firth** | Summer edf = 1.44, p < 0.05 | None edf = 0.0, p > 0.05 | None edf = 0.0, p > 0.05 | Summer, edf = 3.65, p < 0.05 | Summer edf = 2.56, p < 0.05 |
| **Northeast** | Summer  edf = 2.49, p < 0.05 | None edf = 0.0, p > 0.05 | None edf = 0.0, p > 0.05 | Spring, edf = 3.64, p < 0.05 | None edf = 0.0, p > 0.05 |
| **Forth and Tay** | Autumn edf = 3.61, p < 0.05 | None edf = 0.0, p > 0.05 | None edf = 0.0, p > 0.05 | Spring edf = 2.99, p < 0.05 | None edf = 0.0, p > 0.05 |

*Regions are listed in their geographical position, starting in the South West, and continuing clockwise to the South East. Colour key: red = summer peak, orange = autumnal peak, blue = winter peak, green = spring peak, grey = no discernible peak.

Supplementary Figures


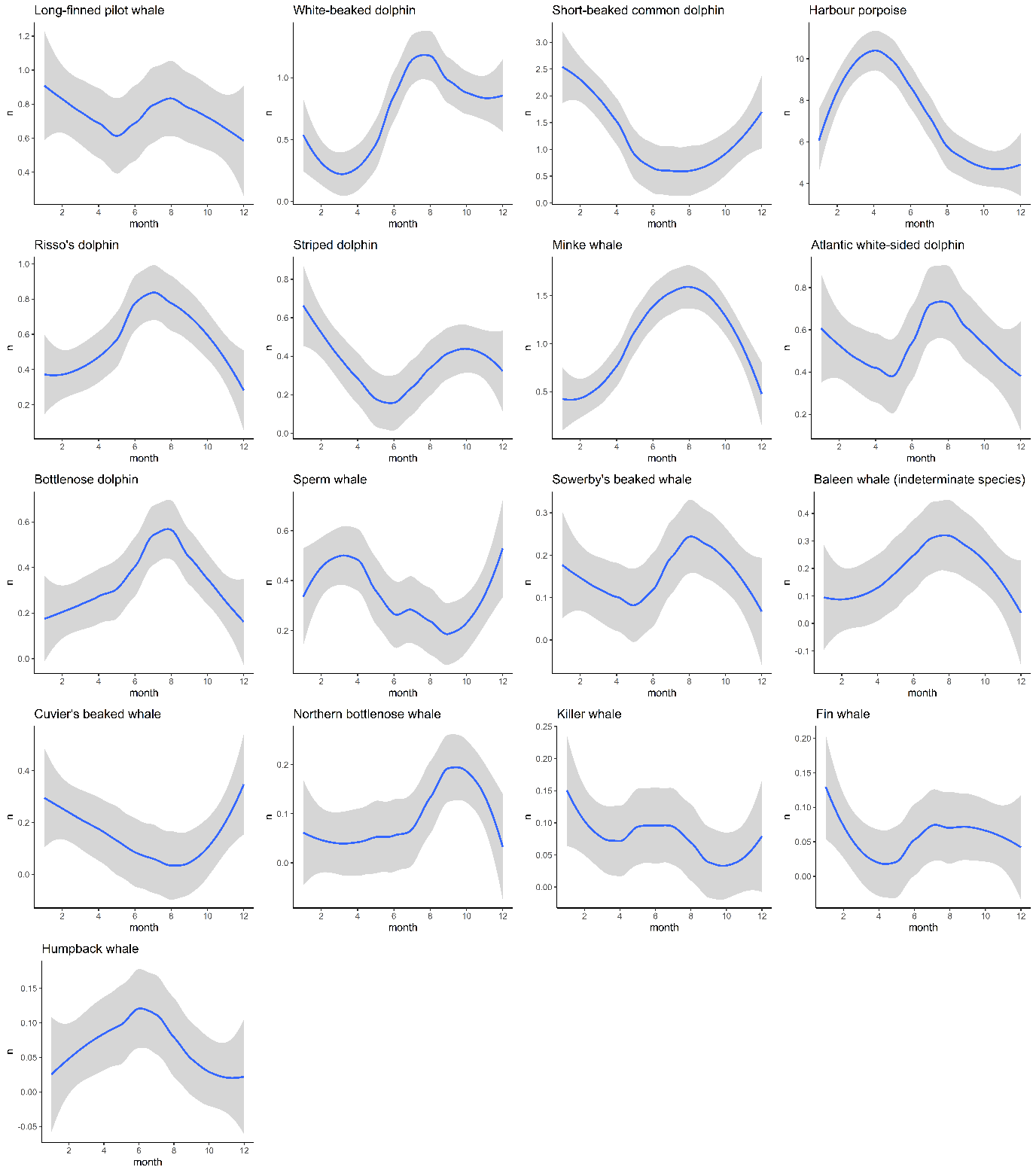


Figure S1: Seasonal trends of all 17 cetacean species stranding rates in Scotland. Lines of best fit were established using method loess to allow for flexibility of trends.


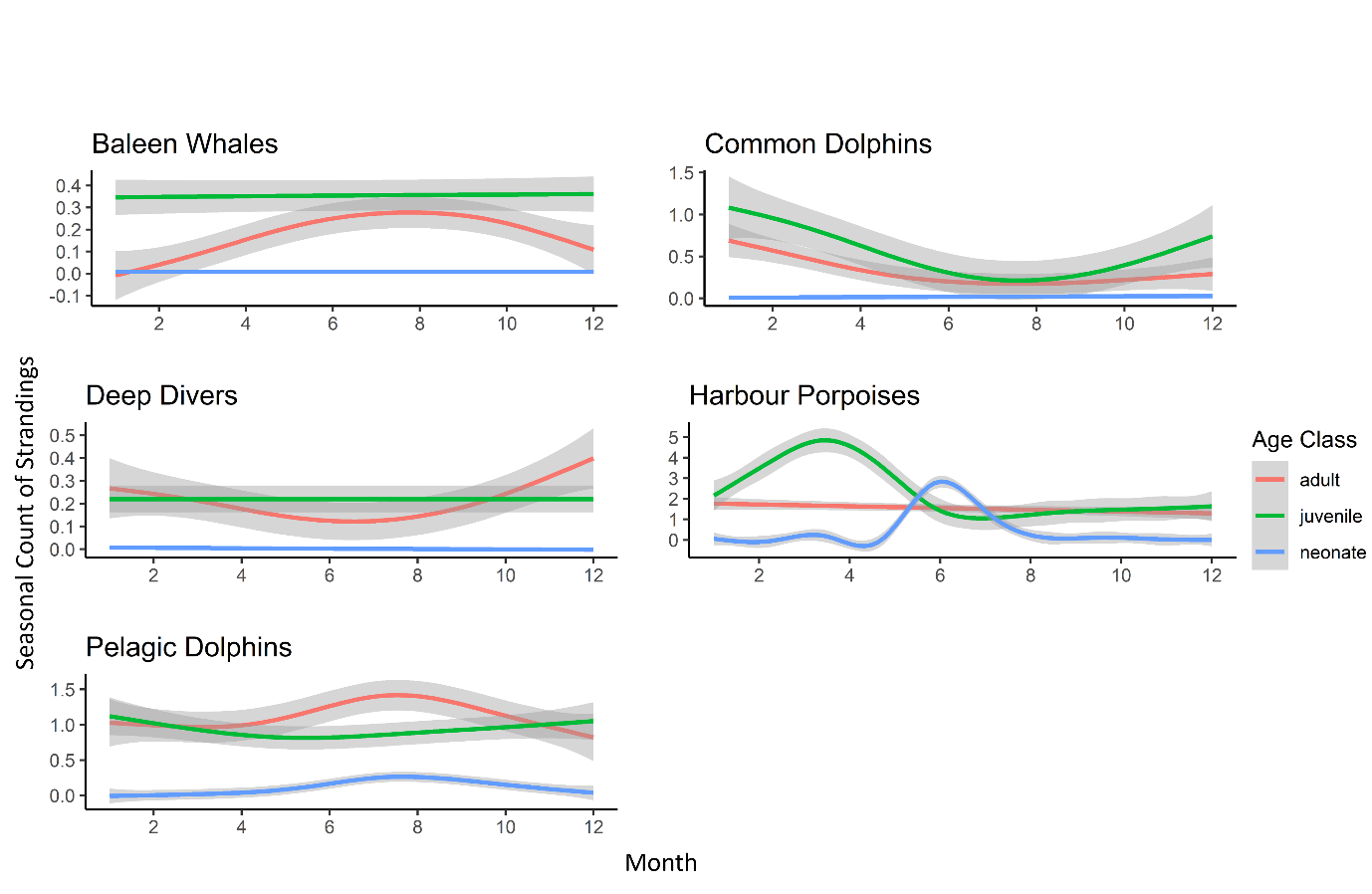


Figure S2: Age specific seasonal trends of stranding rates for five cetacean groups stranding in Scotland. Lines of best fit are fit with method GAM to allow flexibility of trends.


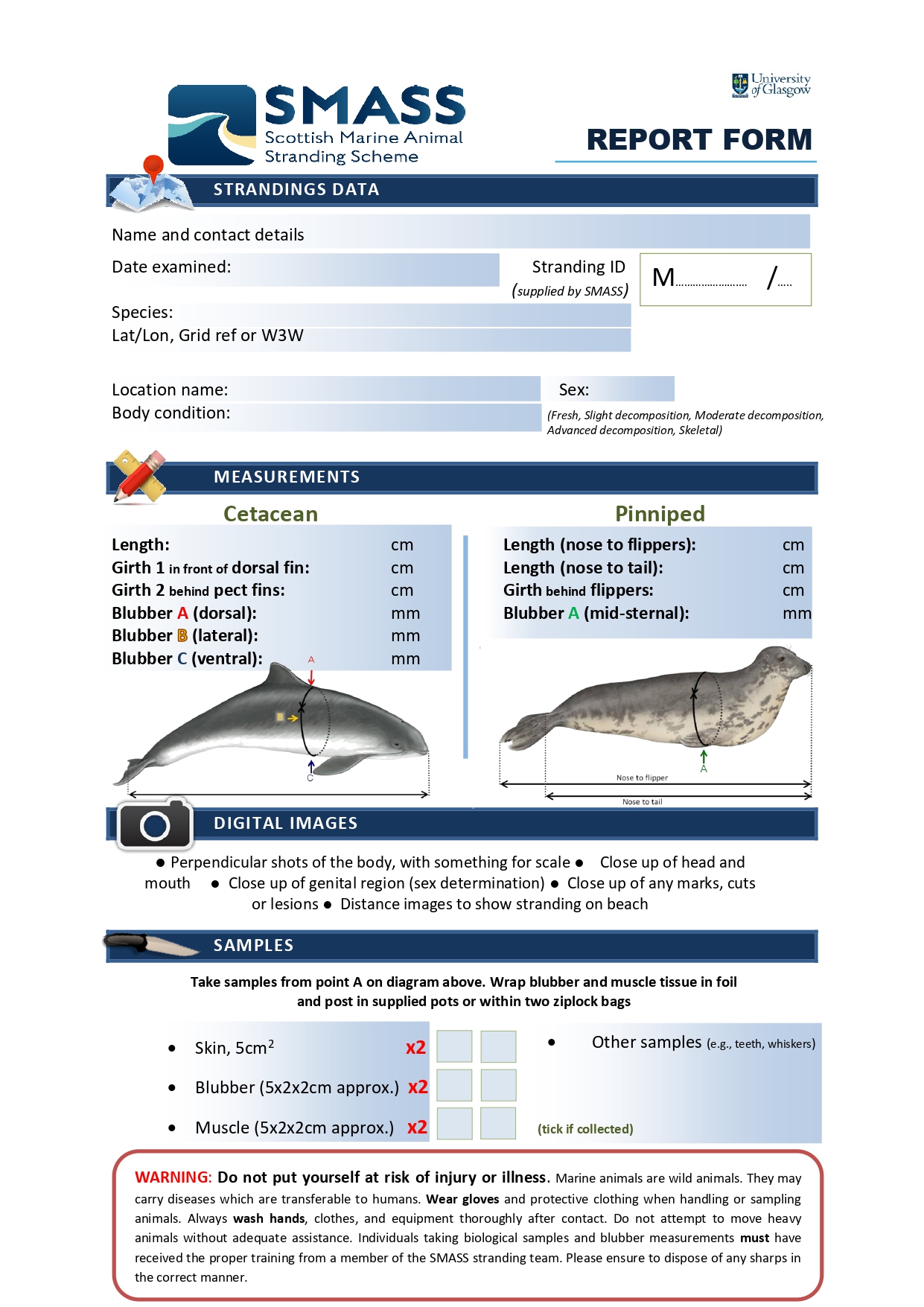


Figure S3: Sampling datasheet used by the Scottish Marine Animal Stranding Scheme (SMASS) to collect Level A data from stranded marine animals in Scotland.
